# The Physician’s Charitable Fund Association

**Published:** 1858-08

**Authors:** 


"*The Physician's Charitable Fund Association.*"—A benevolent society has lately been organized in this city under the above name. The first suggestion in regard to such an organization, was made a few years ago by Dr. James P. White, in his address as retiring president, delivered before the Erie County Medical Society. Taking up this suggestion, a committee was appointed to examine the matter and report. The committee, at the time their report was due, did not think it a favorable time for such an enterprise, and made only a partial report. This committee, however, was continued, and made a report last spring recommending an organization, which was accordingly made, and the following officers were elected: James P. White, President; Austin Flint, Vice-president; Geo. N. Burwell, Treasurer; Jas. M. Newman, Secretary. Eleven of our most prominent medical men were elected trustees.

This association was modeled, in a great measure, after a similar organization in the city of New York. It has for its object the relief of the indigent widows and orphans of medical men, who are members of the association, and of physicians who are poor and disabled. The initiation fee is five dollars, and the yearly dues five dollars. This entitles the family of a member who has paid his dues regularly for two years, to relief from the association, with certain restrictions. We have not the by-laws at hand, and can only state in general terms the more important articles.

We cannot too ardently commend such an association; the labors of the medical man are severe and unremunerative, and it is almost invariably the case that he lives entirely up to his income, and frequently a little beyond it. The anxieties to which a man of feeling is subjected in the practice of medicine, and the all-absorbing nature of his pursuits, leave him but little time to attend to pecuniary matters, and beget an indifference to money which has made physicians proverbially the most improvident class of the community. The families of physicians, then, are peculiarly in need of such an association. They are seldom left in affluence, and not often in comfortable circumstances, and the dangers which surround the path of the doctor, and from which he never shrinks, may deprive his family at any time of their protector, rendering them the more desolate from being reduced often to actual want. It is not probable that the funds of this society will soon be adequate to an extended relief, but it is also improbable that demands will soon be made upon it. The cause is a noble one, and those who have enlisted themselves in it, will not allow it to fail. Every physician should become a member; though he may never expect to be in need of its benefits, yet he will have contributed something to the relief of some one who is not so fortunate, and will have helped to build up an association which will reflect lasting honor on the profession of Western New York.

---

AURORA, Illinois, June 8. 1858.

PROF. AUSTIN FLINT, M. D.,

Dear Sir: I notice a communication in the June number of the Buffalo Medical Journal, from A. M. Leonard, of Lockport, describing a singular occurrence of tumors on the elbow joints. Prof. Hamilton says that symmetrical diseases are known to occur often in erruptive affections, and occasionally in rheumatic and syphilitic affections, but that the case reported is the only example which he has known where an encysted tumor or a true tumor of any kind, has illustrated this curious law of affinities between opposite portions of the body. I have now under treatment the following case, that may have some bearing upon this point:

Was called to see Martin Sort, a stout and healthy German, whom I found with a tumor on the outside of the right thigh, just below and anterior to the trochanter. The tumor was quite painful, and after a few days' use of fomentations, suppurated, after which I lanced it, when it discharged freely and soon began to heal. As soon as this one began to heal, he had another come on the other thigh in exactly the same locality, which acted in every respect just as the first, and is now healing rapidly. He is not aware that he has received any injuries whatever. He is a man of good habits, and free from all constitutional disease.

If you deem the above worthy, you may have it inserted in the Journal, at all events I should like to have it brought before your society, and have the question of sympathy discussed.

Yours in haste,

D. W. YOUNG.

---

*Books Received.*—From Blanchard & Lea: Bucknill & Tuke on Insanity, Lallemand & Wilson on Spermatorrhœa. From Wiley & Halsted: Reid on Ventilation in American Dwellings, Ruskin's Political Economy of Art. From Lindsay & Blakiston: Carnochan, Contributions to Operative Surgery. These publications will receive a more extended notice as soon as we have the space.

*The North American Medico-Chirurgical Review.*—Prof. Richardson, the able associate editor of this valuable periodical having resigned the professorship of anatomy in the Pennsylvania Medical College, and removed to New Orleans to accept a similar chair in the University of Louisiana, has necessarily dissolved his connection with the above-mentioned Journal, and his place has been supplied by Dr. Samuel W. Gross, a son of the eminent senior editor. We have known Dr. Gross, jr., long and well, and it gives us most unfeigned pleasure to welcome him to the editorial fraternity. His writings, which have occasionally appeared in the Review, have given abundant proof of his qualifications for the position which he has assumed, and we predict for him a career not only of usefulness, but eminence in his profession. The public position of the son of so eminent a man as Prof. Gross, while it enjoys the advantage of the reflection of a great name, calls forth all the talent and industry of a more than ordinary mind. We are confident that the new associate editor is in every way capable of sustaining himself under such circumstances, though much will be expected of him, he will be able to do much. He carries with him our warmest congratulations and wishes for his future success.

---

*Appointment of Prof. Flint in the New Orleans School of Medicine.*—It will be perceived on reference to the advertising sheet, that Prof. Flint has been honored by an appointment to the chair of *Clinical Medicine and Auscultation and Percussion*, in the New Orleans School of Medicine. The duties of this chair will commence on the fifteenth of November. In the mean time, Prof. F. will remain at Buffalo, and he expects to return from New Orleans in February. His connection with the school at Buffalo is not dissolved, although his abscence during the greater part of the winter will preclude in a great measure, if not entirely, his participation in the courses of instruction in the latter institution.

---

A new medical journal has just appeared at Athens, Greece, entitled in Greek, "Journal of Medicine." It is to appear once a week, and is edited by Drs. Anagnostakis and Aphendoulis. This makes three medical publications which are now printed in Athens, the two others being monthly in their issue, and are called "The Medical Bee," and "The Esculapian."—*American Medical Monthly.*

*Corrections.*—In the reports of the proceedings of the last meeting of the American Medical Association, as contained in the different medical journals, the motto affixed to the prize essay on the "Clinical Study of the Heart-sounds in Health and Disease," is quoted as follows: *Clinica clinice demonstrandum.* The author of the essay desires us to say that he is not responsible for the bad Latinity of this quotation. As affixed to the essay, it is *Clinica clinice demonstranda.*

---

We wish to apologize for an oversight in sending bills to some subscribers who were not indebted to us, but had paid Prof. Hunt for the whole or part of Vol. XIV. Those who have paid are duly credited, but bills were made out for all subscribers, not recollecting that there were any on the advance list.

